# Reply to: Revisiting life history and morphological proxies for early mammaliaform metabolic rates

**DOI:** 10.1038/s41467-022-32716-z

**Published:** 2022-09-23

**Authors:** Elis Newham, Pamela G. Gill, Michael J. Benton, Philippa Brewer, Neil J. Gostling, David Haberthür, Jukka Jernvall, Tuomas Kankanpää, Aki Kallonen, Charles Navarro, Alexandra Pacureanu, Kelly Richards, Kate Robson Brown, Philipp Schneider, Heikki Suhonen, Paul Tafforeau, Katherine Williams, Berit Zeller-Plumhoff, Ian J. Corfe

**Affiliations:** 1 School of Engineering and Materials Science, Queen Mary University of London, London, UK; 2 School of Earth Sciences, University of Bristol, Bristol, UK; 3 Earth Sciences Department, Natural History Museum, London, UK; 4 School of Biological Sciences, University of Southampton, Southampton, UK; 5 Institute of Anatomy, University of Bern, Bern, Switzerland; 6 Institute of Biotechnology, University of Helsinki, Helsinki, Finland; 7 Ecology and Genetics Research Unit, University of Oulu, Oulu, Finland; 8 Department of Physics, University of Helsinki, Helsinki, Finland; 9 European Synchrotron Radiation Facility, Grenoble, France; 10 Oxford University Museum of Natural History, Oxford, UK; 11 Department of Anthropology and Archaeology, University of Bristol, Bristol, UK; 12 Department of Engineering Mathematics, University of Bristol, Bristol, UK; 13 Bioengineering Science Research Group, Faculty of Engineering and Physical Sciences, University of Southampton, Southampton, UK; 14 High-Performance Vision Systems, Center for Vision, Automation & Control, AIT Austrian Institute of Technology, Vienna, Austria; 15 School of Biological Sciences, University of Portsmouth, Portsmouth, UK; 16 Institute of Metallic Biomaterials, Helmholtz-Zentrum hereon GmbH, Geesthacht, Germany; 17 Geological Survey of Finland, Espoo, Finland

**Keywords:** Palaeontology, Animal physiology

**replying to** S. Meiri & E. Levin *Nature Communications* 10.1038/s41467-022-32715-0 (2022)

In an article examining the physiology of Early Jurassic mammaliaform stem-mammals, we used proxies for basal and maximum metabolic rate, providing evidence that two key fossil mammaliaforms had metabolic rates closer to modern reptiles than modern mammals^1^. Meiri and Levin^2^ questioned the use of our proxy for basal metabolic rate – terrestrial species maximum lifespan in the wild. Here, we explore the evidence behind these differences in viewpoint, and rebut specific points raised by these authors.

The principal point of contradiction between our interpretation of early mammaliaform physiology^1^ and Meiri and Levin^2^ is based on conflicting results for the physiologies of extant mammals between our study^1^ and that of Stark et al.^3^ We found a significant, negative relationship between maximum wild longevity and mass-specific basal metabolic rate (BMR) among extant terrestrial mammals and non-avian reptiles, using phylogenetic generalised least squares (PGLS) regression analysis^1^. However, Stark et al.^3^ similarly analysed a large sample of extant tetrapods and suggested that BMR does not correlate with longevity, across either all tetrapods, or within the amphibian, reptile, bird, and mammal clades. Meiri and Levin^2^ used these findings to question our estimations of low BMRs compared to extant mammals for the mammaliaforms *Morganucodon* and *Kuehneotherium*, based on their long lifespans relative to their size.

## Underlying reasons for conflicting results

We believe the reason for these conflicting results is the differing treatment of extant animal data (body mass, lifespan, and BMR). Specifically: a) Stark et al.^3^ include terrestrial, flying/gliding and marine taxa, whereas we include only terrestrial taxa; b) Stark et al.^3^ pool captive and wild data whereas we analyse them separately and concentrate on wild extant data for comparison with our fossil mammaliaforms; and c) in some analyses, Stark et al.^3^ pool mammals and birds into an ‘endothermic’ sample to be compared to a pooled ‘ectothermic’ sample (non-avian reptiles and amphibians), whereas we compare only terrestrial mammals against non-avian reptiles. Our data treatment was based on a) known exceptions to the relationship between body mass, longevity and metabolic rates (MRs) for flying and marine taxa (whose environments allow, or require, considerably lower or higher body masses, respectively, than terrestrial taxa^4–6^); b) known differences between maximum wild versus captive lifespans of vertebrate taxa (Newham et al.^1^ and references therein,^7^); and c) considerably different evolutionary histories, and resulting physiologies, of individual clades beyond an endotherm/ectotherm dichotomy.

Both studies^1,3^ identify these factors, but deal with them differently. We^1^ limited our extant sample, comparing terrestrial fossil mammaliaforms to terrestrial taxa and analysing wild/captive extant data separately, whereas Stark et al.^3^ include flying and marine taxa and pool wild and captive data. While they accounted for wild versus captive lifespans as a predictor in a multivariate model including body mass, sample size and “metabolic rate comparison” (a binary proxy indicating endothermy versus ectothermy), they did not include these factors when analysing BMR in PGLS regressions between longevity, body mass and BMR (Stark et al.^3^ Table 1). Although they reported nonsignificant differences between wild and captive mammal lifespans (though their reported *p* = 0.04 (Stark et al.^3^ Appendix S3) would seem to indicate significance), we do not consider this a fair test. Wild and captive data for the same taxa are not compared, and wild/captive sample sizes are highly variable between and within the groups considered.

## Data reanalysis

To test these methodological differences, we reanalysed mammal data from Stark et al.^3^ In contrast to the nonsignificant correlations between longevity and a combined PGLS model of BMR and body mass found by Stark et al.^3^ for their full mammal dataset (*n* = 405 including flying/gliding and marine taxa; *p* = 0.23), we find significant correlations for both their wild mammal sample (*n* = 114, p = 0.0056) and their terrestrial wild mammal sample (*n* = 18; *p* = 0.0034) when using their PGLS model (Supplementary Data 1-4). We further applied this model to our data for wild terrestrial mammals^1^ to account for the effect of body mass on the relationship between lifespan and BMR more stringently, as suggested by Meiri & Levin^2^, and also find a significant longevity: BMR correlation (*n* = 117; *p* < 0.0043, Supplementary Data 5-6). PGLS modelling of the data of Stark et al.^3^ with wild versus captive origin, logged body mass, and logged BMR regressed against logged maximum lifespan, shows taxa with wild lifespan data have significantly longer lifespans than captive taxa (*n* = 1062, *p* = 0.0106), the opposite finding to previous studies^1,7^ (Supplementary Data 7-8). We consider this to be due to the dominance of long-lived marine mammals and bats in their wild sample^3^. Jointly, these results highlight the effects of pooling taxa with disparate ecologies and known physiological extremes^4–6^, and not accounting correctly for wild versus captive lifespans^7^. Comparison of Akaike information scores suggests that the addition of an interaction term for each model does not improve their fit beyond the loss of explanatory power created by addition of another explanatory variable, apart from the PGLS model including data origin (significant effect of sample origin retained: *p* = 0.0007) (Supplementary Data 1-8).

Our finding that significant correlations exist between maximum lifespan and BMR among wild terrestrial mammals is upheld in the data of both Newham et al.^1^ and Stark et al.^3^, regardless of PGLS method used. We therefore consider it valid to use PGLS regression between wild longevity and BMR to make our predictions of the physiological status of early mammaliaforms. Their^3^ findings of non-significant differences between lifespans of the pooled mammal/bird ‘endotherm’ grouping and amphibian/reptile ‘ectotherm’ grouping, but significantly longer lifespans of non-avian reptiles than mammals, supports our suggestion that independent treatment of groups with strongly divergent evolutionary, ecological, and physiological histories, such as birds and bats, is more informative than broad grouping in such analyses^8^. Further, while body mass correlates more strongly with both lifespan and BMR than either of the latter factors correlate with each other (as consistently reported for mammals, birds, and non-avian reptiles^9–11^), this does not preclude the use of longevity data to estimate BMR, provided sufficient control is placed on the sample studied, the methodology, and the nature of the predictions.

Regardless of the causes of the relationship (see Box 1), a significant PGLS regression between maximum wild lifespan and BMR in extant terrestrial mammals, maximum wild lifespan and resting metabolic rate in extant terrestrial non-avian reptiles (RMR; synonymous with BMR in mammals), and significant separation between the two regressions using phylogenetic ANCOVA, allowed us to confidently predict BMR/RMR for our mammaliaforms using these regressions^1^.

This relationship has been upheld here when directly employing body mass in our PGLS model, as per Stark et al.^3^, as opposed to regressing mass-specific BMR. The longevities estimated for both fossil taxa are significantly higher than the wild lifespans of extant terrestrial mammal of similar body mass in our data. Of the 36 mammal species highlighted by Meiri & Levin^2^ in the data of Stark et al.^3^ with both comparable size to the mammaliaforms (10–33 g) and lifespans longer than nine years^1^, 35 are bats, which we argue above should be treated independently. The single terrestrial species (*Calomyscus bailwardi*, 9.4 years captive lifespan) has a considerably lower lifespan than our estimated captive lifespans of 12.9 and 17.9 years for *Kuehneotherium* and *Morganucodon*, respectively^1^. Given the strong evidence against powered flight or marine ecologies in both fossil taxa, and the known difference in wild and captive mammal lifespans^1,7^, the most parsimonious interpretation of their difference in longevities from living terrestrial mammals is a different BMR.

Box 1 Mechanistic hypotheses for the lifespan/BMR relationship Rather than the ‘rate-of-living’ theory of ageing, we consider the ‘membrane pacemaker hypothesis’ of Hulbert et al.^9^ to be a likely causal explanation for correlations between BMR and longevity. This was developed to overcome the “number of problems associated with presuming a linkage between rate-of-living and maximum lifespan potential” which Hulbert et al.^9^ identified. Their hypothesis is based on finding a direct causal mechanism between metabolic rate (MR) and the proportion of lipid unsaturation within cell membranes, with higher proportions of unsaturation leading to higher rates of oxidative cellular damage, increased MRs, and decreased longevity. Membranes of larger animals are less susceptible to lipid unsaturation than smaller taxa in the same class, correlating with decreased mass-specific MRs and higher longevity^9,13^. Significant differences in membrane composition also correlate with the exceptionally long lifespans of several mammal clades, especially bats^6^.

When providing their reasoning for the long lifespans of our mammaliaform taxa, Meiri and Levin^2^ suggest that both the exceptional lifespans of bats and early mammaliaforms are predominantly due to reduced predation. Reduced predation is not the only parameter leading to increased longevity in flying vertebrates – in bats this can also reflect reduced rates of cellular damage^6^. We agree with Meiri & Levin^2^ that predation mortality rates of *Morganucodon* and *Kuehneotherium* are currently unknown, but it does not necessarily follow that these were lower than modern rates. A variety of carnivorous lepidosaurs and archosaurs lived coevally with *Morganucodon* and *Kuehneotherium*^14^, as well as a considerably larger morganucodontid mammaliaform^15^ which from tooth shape and size was likely carnivorous^16^.

## Summary

In conclusion, our analyses show that the exclusion of captive data, marine mammal, flying/gliding mammal and bird data from our study of longevity in early terrestrial mammaliaforms is justified, and likely the key reason for the differences in the relationship between BMR and longevity between our study^1^ and those cited by Meiri & Levin^2,3^. We also note that both studies, despite different datasets and methodologies, show that reptiles live longer than mammals, when body size is accounted for. We argue that this result, the significant PGLS regressions found between maximum wild lifespan and BMR/SMR in extant terrestrial mammals and non-avian reptiles, and the results of MMR estimation from femoral blood flow^1^, provide valid tools for estimating metabolic rates in terrestrial mammaliaforms (Box 2). We emphasize that our methodology and data were chosen to provide a biologically meaningful framework for our specific question. We confirm that these methods are valid and support our conclusion that the fossil mammaliaforms *Morganucodon* and *Kuehneotherium* had both basal and maximal metabolic rates outside the range of modern mammals, and that full modern mammalian endothermy had yet to evolve in the Early Jurassic^1,12^.

Box 2 Further rebuttals to specific points We used a second metabolic proxy to support our results^1^ - *Q*_*i*_, an index for relative blood flow to the femur that is correlated with maximum metabolic rate (MMR)^17^. Meiri & Levin^2^ suggest our use of a different method (μCT) for measuring nutrient foramen area^1^, compared to direct calliper measurement or digital optical microscopy^17^, may have resulted in the relatively high *Q*_*i*_ values of small mammal species, and hence *Morganucodon* appearing closer to reptiles. μCT and digital optical microscope foramen measurements have been shown to produce insignificantly different results^18^. Further, while μCT was necessary to overcome issues of sediment infill of foramina in fossils, and small size and oblique penetration angles in extant mammals, even if the relatively high *Q*_*i*_ values of our 11 additional mammal species were due to the μCT method, this would equally apply to the *Q*_*i*_ of *Morganucodon*. The relative difference in their position, with *Morganucodon* having considerably lower *Q*_*i*_ than all small mammals, would be conserved, and indeed, if adjusted downwards in *Q*_*i*_ value, *Morganucodon* would be placed within the MMR range of modern reptiles. Meiri & Levin^2^ also questioned whether the ‘phylogenetically clustered’ nature of our additional mammal sample might have made *Morganucodon* appear closer to reptiles. Larger phylogenetic coverage is desirable for future studies, but the suggestion of clustering is undermined by the wide phylogenetic separation of Rodentia and Eulipotyphla in our sample and the lack of phylogenetic signal in *Q*_*i*_ calculated using PGLS regression (lambda < 0.001, Newham et al.^1^ Methods).

Meiri & Levin^2^ highlight several studies we cited^1^ that they consider better proxies of metabolic rates than maximum longevity, stating nasal turbinates^19^ operated “as heat exchange surfaces in *Morganucodon*, as they do in extant (endothermic) mammals”. However, Crompton et al.^19^ actually strongly support our results, stating that, while cartilaginous turbinates were likely present in *Morganucodon* and used for heat exchange, elevated endothermic metabolic rates only originated with the evolution of ossified respiratory turbinals in Middle Jurassic crown mammals and their closest relative. These authors^19,20^ suggested further that high metabolic rates, endothermy and ossified respiratory turbinals were all lacking in *Morganucodon* and closely related mammaliaforms. More generally, our reason for citing these varied studies was to show the wide range of times suggested for the origin of mammalian endothermy with different methods and the reinterpretation of many of the characters previously associated with endothermy^1,21^ (and see Newham et al.^12^).

Finally, Meiri & Levin^2^ open their comment with three ‘key mammalian characteristics’ they claim that *Morganucodon* and *Kuehneotherium* possessed. None of these characters is a key characteristic associated with the origin of crown group Mammalia: a) multi-cusped teeth considerably predate these Late Triassic/Early Jurassic mammaliaforms^20^ and are widely found in reptiles^22^, whereas diphyodonty and notably complex occlusion first appear in such mammaliaforms^1,23^; b) as noted above, ossified respiratory turbinates, as found in extant mammals, are not present in *Morganucodon* nor *Kuehneotherium* (but do appear outside crown group Mammalia^19,20^); and c) Harderian glands are universal throughout tetrapods, while their association with grooming and pelage maintenance is unknown beyond rodents^24^ and cannot be used to infer the presence of fur in the mammaliaforms *Morganucodon* and *Kuehneotherium*.

## Methods

All physiological data (maximum lifespan, data origin, mean body mass, basal metabolic rate) used for analyses originate from samples of extant mammals in the published datasets of Stark et al.^3^ and Newham et al.^1^ Phylogenetic generalised least squares regression analysis (PGLS) was performed in the “R” statistical environment with the “ape”, “nlme”, “geiger”, “ggpubr”, “lmtest”, “phytools”, “plyr”, “car”, “fmsb”, “FSA”, “ggplot2”, and “caper” packages installed. Phylogenetic data was provided by G. Stark, which was then imported into two PGLS modelling techniques used to inform least squares regression between multiple factors with phylogenetic data. The first technique input phylogenetic and physiological data into the “corPagel” covariance structure for the “gls()” function to produce phylogenetically informed regression models between physiological metrics^1^. The second technique, following the methodology outlined in Appendix S4 of Stark et al.^3^, input phylogenetic and physiological data into the “pgls()” function with the lambda variable set to “ML” to optimise branch length transformations. Each model was analysed both with and without an interaction term, and the effect of the interaction term assessed by comparing the Akaike Information Criterion (AIC) score of each model.

### Reporting summary

Further information on research design is available in the Nature Research Reporting Summary linked to this article.

## Supplementary information

Description of Additional Supplementary Files

Supplementary Data 1-8

Reporting Summary

## Data Availability

Physiological and phylogenetic data reanalysed from Newham et al.^1^ are from online databases of the Max Planck Institute (https://www.demogr.mpg.de/longevityrecords/0203.htm), an online Ecological Archives database (http://www.esapubs.org/archive/ecol/E084/094/metadata.htm), the AnAge database (https://genomics.senescence.info/species/), the VertLife online project (https://vertlife.org) and the literature (references in Supplementary Data 3 of Newham et al.^1^) and were provided in Supplementary Tables, as Supplementary Data files, and as a part of the Source data provided with Newham et al.^1^ Physiological and phylogenetic data reanalysed from Stark et al.^3^ were provided in Supporting Information Appendix S1 of Stark et al.^3^
