# Supplementary material for: Reply to: Revisiting life history and morphological proxies for early mammaliaform metabolic rates
Source: Nat Commun. 2022 Sep 23;13:5564. doi: 10.1038/s41467-022-32716-z (PMC9508248; doi:10.1038/s41467-022-32716-z)
Supplement: Supplementary file 1 — Description of Additional Supplementary Files [file 41467_2022_32716_MOESM1_ESM.pdf]

Description of Additional Supplementary Files

File Name: Supplementary Data 1
Description: One-sided phylogenetic generalised least squares (PGLS) regression model parameters assessing the relationship between maximum lifespan and the combination of logged mean body mass and logged BMR for the wild mammal data of Stark et al 2020[3] (no adjustments made for multiple comparisons).

File Name: Supplementary Data 2
Description: One-sided phylogenetic generalised least squares (PGLS) regression model parameters assessing the relationship between maximum lifespan and the combination of logged mean body mass and logged BMR for the wild mammal data of Stark et al 2020[3], plus their interaction term (no adjustments made for multiple comparisons).

File Name: Supplementary Data 3
Description: One sided phylogenetic generalised least squares (PGLS) regression model parameters assessing the relationship between maximum lifespan and the combination of logged mean body mass and logged BMR for the wild terrestrial mammal data of Stark et al 2020[3] (no adjustments made for multiple comparisons).

File Name: Supplementary Data 4
Description: One-sided phylogenetic generalised least squares (PGLS) regression model parameters assessing the relationship between maximum lifespan and the combination of logged mean body mass and logged BMR for the wild terrestrial mammal data of Stark et al 2020[3] and their interaction (no adjustments made for multiple comparisons).

File Name: Supplementary Data 5
Description: One-sided phylogenetic generalised least squares (PGLS) regression model parameters assessing the relationship between maximum lifespan and the combination of logged mean body mass and logged BMR for the wild terrestrial mammal data of Newham et al (2020)[1] (no adjustments made for multiple comparisons).

File Name: Supplementary Data 6
Description: One-sided phylogenetic generalised least squares (PGLS) regression model parameters assessing the relationship between maximum lifespan and the combination of logged mean body mass and logged BMR for the wild terrestrial mammal data of Newham et al (2020)[1] and their interaction (no adjustments made for multiple comparisons).

File Name: Supplementary Data 7
Description: One-sided phylogenetic generalised least squares (PGLS) regression model parameters assessing the relationship between maximum lifespan and the combination of logged mean body mass, logged BMR, and sample origin (wild versus captive) for the mammal data of Stark et al 2020[3] (no adjustments made for multiple comparisons).

File Name: Supplementary Data 8
Description: One-sided phylogenetic generalised least squares (PGLS) regression model parameters assessing the relationship between maximum lifespan and the combination of logged mean body mass,

logged BMR, and sample origin (wild versus captive) for the mammal data of Stark et al 2020[3], and their interaction (no adjustments made for multiple comparisons).
